# Visceral Leishmaniasis Following A+AVD Treatment in a Patient with Classical Hodgkin’s Lymphoma: A Case Report and Review of the Literature

**DOI:** 10.3390/jcm13195756

**Published:** 2024-09-27

**Authors:** Daniela Estefania Banegas, Alessia Moioli, Eleonora Santoni, Erica Tagliavini, Francesca Maria Quaglia, Andrea Bernardelli, Carlo Visco

**Affiliations:** Hematology Unit, Section of Biomedicine of Innovation, Department of Engineering for Innovative Medicine, Verona University, 37134 Verona, Italy

**Keywords:** Hodgkin’s lymphoma, brentuximab–AVD, A+AVD, visceral leishmaniasis, splenomegaly

## Abstract

We present the case of a 43-year-old Caucasian man who developed visceral leishmaniasis (VL) following treatment with a combination of brentuximab vedotin and doxorubicin, vinblastine, and dacarbazine (A+AVD) for advanced-stage classical Hodgkin’s lymphoma (cHL). The patient initially showed a favorable response to the treatment, but shortly after completing six cycles, he experienced recurrent fever, splenomegaly, and severe anemia. Extensive infectious disease evaluations led to a diagnosis of VL, confirmed by PCR testing. The patient was treated with amphotericin B, resulting in full clinical recovery. In addition to presenting this rare case, we conducted a full review of the literature on VL in the context of hematological disorders, including non-Hodgkin’s lymphoma, splenic marginal zone lymphoma, and other lymphoproliferative diseases. This review highlights the increasing prevalence of VL in immunocompromised individuals, particularly those undergoing treatments like chemotherapy or immunotherapy, and underscores the importance of considering VL in differential diagnoses when such patients present with persistent fever and splenomegaly.

## 1. Introduction

Classical Hodgkin’s lymphoma (cHL) has a global annual incidence of 100,000 cases. Annually, approximately 8540 new cases are reported in the United States [1]. The reported incidence rate in the European Union is 2.3 per 100,000 inhabitants [2].

cHL neoplastic cells are large dysplastic mononuclear and multinucleate cells, commonly known as Reed–Sternberg cells. A combination of mature non-neoplastic inflammatory cells surrounds these abnormal cells. This tumor microenvironment (TME) can expand regulatory T-cells, thus inhibiting CD8-positive cells and repolarizing tumor-associated macrophages. Consequently, the interaction between lymphoma cells and the TME leads to immune deficiency in the affected subjects [3]. Growing interest in the biology and microenvironment of cHL has led to the adoption of innovative and effective strategies, including targeted therapies, immunotherapy, and cell therapy [4]. For example, brentuximab vedotin (A), an anti-CD30 antibody–drug conjugate that was initially approved for relapsed and refractory cHL, has been recently incorporated into the AVD regimen (A+AVD), with a reported survival advantage compared to the traditional AVD plus bleomycin scheme (ABVD) in advanced-stage cHL [5]. Indeed, the ECHELON-1 study has established superior efficacy in terms of the modified progression-free survival of A+AVD over ABVD in the treatment of patients with advanced-stage cHL. All secondary efficacy endpoints were in favor of A+AVD, including the combined risk of progression, death, or noncomplete response, as well as the use of subsequent anticancer therapy at two years [5]. The most recent update of the trial reported a 6-year overall survival advantage of A+AVD (93.9%) compared to ABVD (89.4%) [1]. However, the A+AVD regimen has also been linked to an increased risk of infection compared to the ABVD backbone, indicating that innovative treatments should always require careful consideration of potential new and rare adverse events.

Phlebotomine sand flies are the primary vectors responsible for transmitting leishmaniasis [6]. Symptoms can vary from ulcerative skin lesions to deforming mucosal lesions (tegumentary leishmaniasis) or liver and spleen hypertrophy, which is the case of visceral leishmaniasis (VL). Visceral leishmaniasis (VL) can be caused by two main species: L. donovani and L. infantum. Globally, about 500,000 new cases of VL occur worldwide every year. If not appropriately treated, over 95% of patients affected by VL will die [7]. In Italy, between 2009 and 2016, 1126 cases of VL were identified, resulting in an incidence rate (IR) of 2.43 cases per 10^6^ persons per year [8]. This infection caused by parasites can emerge as an opportunistic disease after a recent illness or as the reactivation of an underlying condition. The clinical picture is characterized by prolonged fever, weight loss, anemia, and hepatosplenomegaly [7].

Leishmaniasis is an intracellular infection that adversely impacts the activation and functionality of macrophages and dendritic cells, allowing malignant cells to evade immune destruction. Additionally, chronic leishmaniasis infections result in CD4 lymphopenia and a reduced CD4/CD8 ratio. The noticeable immunosuppression observed during VL is believed to be influenced by elevated levels of regulatory T-cells, leading to a compromise in adaptive immune responses [9].

Leishmaniasis has rarely been described in patients with cHL. The case presented here is a rare association of VL and cHL in the setting of post-A+AVD chemo-immunotherapy. We also provide a comprehensive review of VL cases in other lymphoproliferative disorders.

## 2. Case Description

**Figure 1:**
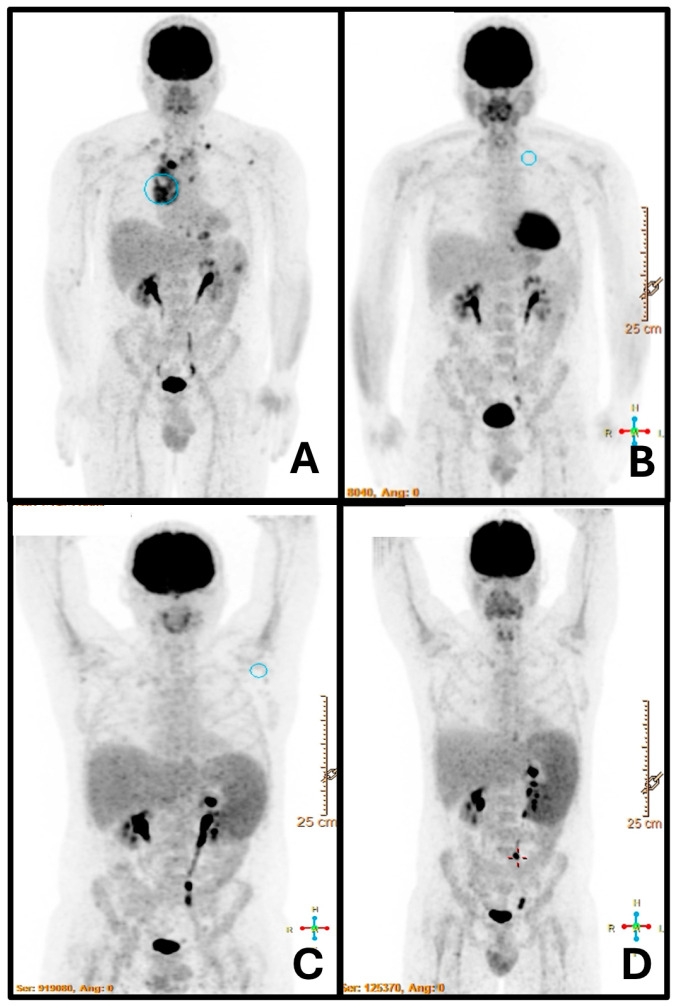
PET-TC at cHL diagnosis (**A**): increased glycolytic metabolism observed in multiple lymph nodes, precisely above the diaphragm, at the scapular level, and a notable lesion in the spleen without splenomegaly. After completing two cycles of immuno-chemotherapy (**B**), a complete response was achieved, and no evident lesions were observed at the spleen level. PET-CT performed one month post-treatment (**C**) revealed a homogeneous splenomegaly of 18 cm with no focal lesions (an abdominal ultrasound confirmed the absence of spleen lesions). Two months later, imaging confirmed an increase in splenomegaly measuring 20 cm, without any other findings (**D**), when a diagnosis of VL was established.

A 43-year-old gentleman was diagnosed with nodular sclerosis stage IV cHL with focal involvement of the spleen and bone marrow in February 2023 (Figure 1A). According to his medical history, he had undergone a total thyroidectomy 12 years before due to a papillary tumor. Additionally, he was known to be a frequent traveler. He visited Argentina (2006) and Brazil (2010), with his most recent trip being to Australia in 2022.

The patient underwent six cycles of A+AVD treatment, achieving an early treatment response, as documented by positron emission tomography (PET-TC) after two cycles (Figure 1B). Treatment was completed in August 2023. One month later, a PET-CT scan conducted post-treatment revealed a new onset of splenomegaly, which measured 18 cm and had an increased uptake of 18FDG without associated focal lesions or other abnormalities (Figure 1C). This finding contrasted with the imaging conducted during diagnosis, which revealed spleen lesions without splenomegaly. Simultaneously, our patient reported fever and self-limited diffuse erythema for several days. At the same time, his two-year-old child was diagnosed with erythema infectiosum caused by Parvovirus B19 (fifth disease), and for this reason, the same disease was initially suspected. A physical examination revealed splenomegaly, confirmed by abdominal ultrasound (splenic span of 18 cm). Laboratory tests indicated mild anemia (hemoglobin Hb: 11 g/dL), with a leukocyte count of 2400 cells/mm^3^, a neutrophil count of 1200 cells/mm^3^, and a C-reactive protein level of approximately 95 mg/L. Serum levels of immunoglobulins were not recorded in our case. Extensive microbiological tests were conducted, including urine analysis, chest X-rays, and blood cultures. Subsequently, the IgM antibody turned positive for Parvovirus B19, Toxoplasma, and Enterovirus. Parvovirus B19 DNA was detected by reverse transcriptase–polymerase chain reaction (RT-PCR), with a count of 658,439 copies/mL. As part of the initial management, we administered granulocyte colony-stimulating factor (G-CSF) and intravenous immunoglobulin therapy, which resulted in modest clinical improvement.

Although Parvovirus DNA declined to undetectable levels, fever persisted, associated with a weight loss of 8 kg in two months, and the blood counts were severely impaired in terms of anemia and leukopenia (Hb: 8.1 g/dL, WBC: 1650 cells/mm^3^). Considering the potential for cHL relapse, another PET-CT scan was performed, confirming splenomegaly (SUV max: 6.7) with no other apparent abnormalities (Figure 1D). This was supported by ultrasound, which revealed increased splenomegaly to 20 cm. In agreement with our infectious disease specialists, given the clinical and laboratory deterioration of the patient, RT-PCR for leishmaniasis was performed on peripheral blood. The test turned positive, finally establishing a diagnosis of VL.

The patient was started with amphotericin B at 4 mg/kg for five days, followed by an additional five weekly doses of 4 mg/kg as part of maintenance therapy. This treatment resulted in a sudden resolution of all symptoms. The abdominal ultrasound showed a significant reduction in splenomegaly with normalization of spleen diameter one month after completion of the therapy. Three months later, the patient was asymptomatic, with normal blood counts. Approximately one year later, he is doing well and is in active follow-up for his cHL.

## 3. Discussion and Review of the Literature

Leishmaniasis is widely recognized as an opportunistic infection. Sandfly bites transmit leishmaniasis in parts of Asia (primarily India), Africa (mainly Sudan), and South America (primarily Brazil), where, together, there are an estimated half a million cases per year. However, following the global trend of immigration, for the number of populations susceptible to infections due to immunosuppressive factors, co-morbidities, and aging, the disease has demonstrated a significant increase in its incidence in non-tropical countries. Therefore, the potential for leishmaniasis to re-emerge, due to increased vulnerability to new infections or reactivation of inactive infections, is significant and should not be ignored [6]. The elderly population (probably because of immunosenescence), adults medically immunosuppressed by chemical or biological drugs, and adults with malignant tumors, autoimmune disorders, or any signs of immunodeficiency, who live in or travel to endemic countries, are categories particularly at risk [6]. Apart from human immunodeficiency virus (HIV) status, hematological malignancies are the most usual underlying cause of immunodeficiency in patients who develop VL.

The pathogens Leishmania donovani, L. infantum, and L. chagasi are linked to VL [10]. L. infantum is the most prevalent species in the Mediterranean area. In humans, parasites in the amastigote form are typically found intracellularly within the reticuloendothelial system, a flagellate 2–4 μm in diameter (Leishman–Donovan body). Symptoms usually develop after an incubation period of weeks/months. If left untreated, progressive illness can result in death. Asymptomatic stages and relapses suggest that parasites can persist in tissues for a long time prior to the clinical onset of the disease [10].

VL is also known as “kala-azar”, which by medical definition is a chronic, potentially fatal parasitic disease of the viscera, which can particularly affect the liver, spleen, lymph nodes, and bone marrow due to infection by the parasite Leishmania donovani. Kala-azar can be associated with fever, anorexia, fatigue, liver or spleen enlargement, nodes, and bone marrow suppression. The term “kala-azar” originates from India, where it translates to “black fever” in Hindi. VL is also known as Indian leishmaniasis, visceral leishmaniasis, leishmania infection, dumdum fever, black sickness, and black fever [11].

Non-HIV-related VL is progressively appearing in non-tropical countries; this is attributed not only to the increasing number of patients with chronic illnesses but also to the rapid advancement of immune-modulating therapies for the treatment of neoplastic diseases. Various cases of VL in patients with lymphoproliferative disorders have been documented since 1988 [12,13,14,15,16,17,18,19,20,21,22,23,24,25,26] (Table 1). These reports describe VL occurring in several lymphoproliferative disorders, such as cHL, splenic marginal zone lymphoma (SMZL), follicular cell lymphoma, lymphoplasmacytic lymphoma, chronic lymphocytic leukemia, angioimmunoblastic T-cell lymphoma, and T-cell prolymphocytic leukemia. In the majority of them (at least 8 of 12), VL was incorrectly identified as an advancement of the underlying hematological cancer. Although it is difficult to prove this with certainty, VL that arises in non-endemic areas may represent parasite reactivation that took place during episodes of immune system suppression, such as following hematopoietic cell transplantation, as demonstrated in a case involving concomitant VL, lymphoma relapse, and graft failure [27], or as a consequence of a recent acute infection after travel to endemic countries. In the paper by Galith et al., for example, both patients had recently traveled to endemic areas from a non-endemic area (central France) [12].

Healthcare providers should recognize the signs of VL and consider it as part of a differential diagnosis in lymphoma patients. Clinical signs such as splenomegaly, fever, and weight loss, along with laboratory findings like pancytopenia, are frequently nonspecific. In some cases, patients have been reported to develop hypergammaglobulinemia as a result of polyclonal B-cell activation. One article noted that *L. donovani* exacerbates the disease by promoting IL-10 and IFN-I production while also inducing hypergammaglobulinemia [28].

In patients treated with drugs like rituximab or other monoclonal antibodies that interfere with antibody production, serological diagnosis of VL may be missed or delayed. Therefore, a blood or bone marrow polymerase chain reaction (PCR) might be a more suitable diagnostic tool for VL in lymphoma patients.

It has been suggested that leishmaniasis may be implicated in the pathogenesis of some patients who develop SMZL. Indeed, [19,24] some microbiological agents, either bacterial or viral (i.e., hepatitis C virus or helicobacter pylori) have been widely implicated in the pathogenesis of several lymphoid malignancies, including SMZL. Although there might be a possible connection due to persistent antigenic stimulation from microbial agents, leishmaniasis is still far from being implicated in such a scenario. The parasites invade the spleen and stimulate macrophages in the marginal zone, thus sustaining antigenic stimulation and possibly triggering polyclonal B-cell proliferation [24]. We believe that these important aspects should be investigated more deeply in future research.

Finally, a case of VL in a 66-year-old female with a diagnosis of MALT lymphoma in the gastrointestinal tract has been reported [29]. She exhibited severe hemorrhage and perforation of the small intestine. Due to a simultaneous unexplained decreasing platelet count, a bone marrow examination was executed to rule out lymphoma involvement, and, surprisingly, Leishmania donovani bodies were detected. Similar to our report, a lipid formulation of amphotericin B was used for treatment, given at a dosage of 4 mg/kg daily over a five-day period. After suffering from acute renal dysfunction, which is a well-known complication associated with these antifungal agents, the patient recovered and could proceed to systemic chemotherapy with a good response.

The association between cHL and VL appears to be a rare combination. To the best of our knowledge, only four cases (excluding ours) have been described in the literature so far for non-HIV cHL. One additional case from an endemic area of Brazil was reported in lymphocyte-predominant (LP-) HL (case number 5). In all cases, the outcome appeared favorable, provided that an accurate diagnosis was made as early as possible in order to prevent severe complications related to parasite proliferation within the host favored by an immunosuppressed environment. The main characteristics of patients with cHL reported in the literature are summarized in Table 2. The occurrence of both lymphoma and leishmaniasis within the same lymph node was documented in two individuals with HL [9,12]. Still, the majority of the diagnoses were performed on marrow smears or by serology. All patients had undergone cytotoxic chemotherapy except for one patient. VL developed at the time of lymphoma diagnosis in three patients, before diagnosis in one, and during the course of the disease in the remaining cases (LP-HL). The latter study highlights the significance of recognizing VL as a potential mimic and opportunistic infection in HL, even at the stage of lymphoma relapse, where the symptoms and signs of VL can be easily confused with tumor recurrence.

Our case was apparently the first case of VL in a patient treated upfront with a combination therapy different from ABVD for cHL (A+AVD). The range of side effects mediated by cHL traditional chemotherapy (ABVD), compared to novel immunotherapy, including the addition of the antibody–drug conjugate A, poses diagnostic and therapeutic challenges, mainly because several infections were not recorded during the studies that enabled these drugs’ approval. In our particular case, we used the combination A+AVD for the frontline treatment of our patient with advanced-stage HL since the combination was associated with a 23% decrease in the risk of progression compared to ABVD [5] and with a survival advantage in the randomized phase 3 study ECHELON-1. While brentuximab vedotin may induce an immune imbalance that can facilitate infections, and many opportunistic infections are increasingly described after this combination, no leishmaniasis cases were included in the safety registry of the ECHELON-1 study. Nevertheless, the A+AVD combination was associated with an increased risk of febrile neutropenia, and primary prophylaxis with G-CSF was mandatory during the study. Indeed, in the A+AVD arm, 55% of patients experienced an infectious event, and nine died, suggesting that this combination may be associated with a higher risk of infection than ABVD alone [1].

Interestingly, a retrospective study evaluated the incidence and characteristics of infections in patients undergoing treatment with A (with or without chemotherapy) for cHL, non-Hodgkin’s T-cell lymphoma, and anaplastic large-cell lymphoma across 14 Italian hematology centers [33]. The study included 191 patients treated with A. Overall, 12% of these patients experienced one or more infections during their treatment, predominantly pulmonary infections (20%) and CMV/EBV reactivations (20%). The mortality rate was 0.5%. Bacterial infections accounted for 55% of cases, viral infections for 30%, and fungal infections for 15%. No parasite infection was documented during the 335 days covered by this study [12]. No case of VL was reported.

It is well known that VL may present with clinical and computed tomography (CT) examination mimicking lymphoma. CT imaging may not always provide a straightforward differential diagnosis due to the involvement of the lymphoid compartment in both diseases [34]. Since the diagnosis of VL is complicated and often unsuspected, limited knowledge exists regarding the efficacy of nuclear imaging techniques. An important study has been conducted in an attempt to discern the utility of fluorodeoxyglucose positron emission tomography/computed tomography (FDG-PET/CT) in cases of VL [35]. In a retrospective study, the authors examined VL cases diagnosed at Vall d’Hebron University Hospital between 2012 and 2018, focusing on those patients who had undergone FDG-PET/CT scans. Among the 43 patients diagnosed with VL, 4 were subjected to FDG-PET/CT scans. They all presented diffuse splenic uptake of FDG-PET/CT (as was the case of the patient described in the present report; see Figure 1). Lymphadenopathy was not always detected, and bone marrow uptake was seen in two of the patients. A post-treatment FDG-PET/CT in one patient showed a normalization of initial findings (as for our patient). The pattern of spleen involvement described in the literature demonstrated disparate presentations, ranging from “diffusely increased metabolism” to “nodular pattern” or “patchy and granular”. Other common findings included bone marrow uptake and adenopathy. The authors concluded that FDG-PET/CT has the potential to be a valuable tool for diagnosing and monitoring VL. In cases of fever of unknown origin with splenic uptake observed on FDG-PET/CT, VL should be considered. Of course, differential diagnosis is always mandatory due to the low specificity of this diagnostic technique.

## 4. Conclusions

To the best of our knowledge, we describe the first case of VL in a patient treated upfront with A+AVD for cHL. Infectious complications when treating these patients should always be considered, though they can resemble tumor progression or resistance, as in our case. A multidisciplinary approach is warranted in those cases. We believe VL should be ruled out when persistent fever, unexpected cytopenias, or splenomegaly occurs in lymphoma cases, regardless of geographical area or epidemiological risk factors. A blood Leishmania PCR should be carried out in cases with suspected signs.

## 5. Future Directions

With the rise of novel therapies, it is important to recognize that infections affecting our patients are not limited to bacterial or viral sources, but also include parasitic infections. Unfortunately, research on parasitic infections in the context of new immune-cellular therapies is still limited, as these infections are not extensively covered in the clinical studies associated with these new drugs. Therefore, further studies focused on parasitic infections in this context are necessary.

## Figures and Tables

**Table 1:** A review of VL cases among patients with lymphoproliferative disorders (VL: visceral leishmaniasis).

Type of Lymphoma	Studies Related, Year	Time Point of VL Diagnosis	Symptoms of VL	Lymphoma Treatment	Outcome
Splenic marginal zone lymphoma	Vase, 2011 [17]	Before lymphoma diagnosis	Fever, splenomegaly, pancytopenia	Splenectomy + Rituximab	Complete Remission
	Evers, 2014 [18]		splenomegaly, pancytopenia	Splenectomy	Complete Remission
Follicular Lymphoma	Casabianca, 2011 [19]	After lymphoma diagnosis	Splenomegaly, lymphadenopathy	Rituximab + Chemotherapy	Complete Remission then Relapse
Angioimmunoblastic T-Cell	Osakwe, 2013 [26]	After lymphoma diagnosis	Fever, splenomegaly, lymphadenopathy, macular rash	Rituximab + Chemotherapy	Not Available
Lymphoplasmocity Lymphoma	Cencini, 2015 [20]	Not available	Splenomegaly, pancytopenia	Rituximab + Bendamustina	Complete Remission
T-Cell-Prolymphocytic Leukemia	Liao, 2018 [21]	Simultaneous	Weight loss, lymphadenopathy, splenomegaly, fever, pancytopenia	Not Available	Deceased
Lymphocytic Lymphoma	Kalmi, 2020 [12]	After lymphoma diagnosis	Weight loss, lymphadenopathy, splenomegaly, anemia	Rituximab + Bendamustina	Complete Remission
Peripheral T-Cell Lymphoma Not Otherwise Specified	Kalmi, 2020 [12]	After to lymphoma diagnosis	Fever, lymphadenopathy, splenomegaly, pancytopenia	Chemotherapy	Complete Remission then relapse
Chronic Lymphocytic Leukemia (CLL)	Orlandi, 2014 [23]	After lymphoma diagnosis	Splenomegaly, lymphadenopathy	Rituximab + Chemotherapy + Alemtuzumab	Partial remission then relapse
	Nicolas, 2018 [24]	After lymphoma diagnosis	Fever, weight loss, lymphadenopathy, splenomegaly, pancytopenia	Not Available	Not Available
	Zanelli, 2022 [25]	Simultaneous	Fatigue, diarrhea, pancytopenia, splenomegaly	Ibrutinib (after VL treatment)	Complete Remission

**Table 2:** Features and progression of patients with cHL and VL as reported in the literature (cHL: classical Hodgkin lymphoma; VL: visceral leishmaniasis; ABVD: doxorubicin, bleomycin, vinblastine, and dacarbazine).

Studies Related, Year, and Country	Time Point of VL Diagnosis	Samples for the Diagnosis of Leishmaniasis	Outcome	cHL First-Line Treatment
Kumar R, 2011. India [30]	At diagnosis of cHL, pre-chemotherapy	Bone marrow aspirate and serology test	Alive	Chemotherapy not described
Gomes Porto VB, 2022. Brazil [31]	At diagnosis of cHL, pre-chemotherapy	Bone marrow aspirate and serology test	Alive	ABVD
Magnan A, 1991. France [12]	At diagnosis of cHL, pre-chemotherapy	Bone marrow aspirate	Unknown	Not reported
Dereure J, 2003. France [32]	In patient with history of cHL	Bone marrow aspirate and immunofluorescence antibody test	Unknown	Splenectomy and radiotherapy
Domingues M, 2009. Brazil [9]	During treatment for lymphocyte-predominant HL	Lymph node	Alive	ABVD
Tatarelli P, 2018. Italy [27]	During hematopoietic cell transplantation	Bone marrow aspirate and PCR in a sample of peripheral blood	Died +212 days after haploidentical transplantation	Not reported
